# New Hybrid Polyurea-Polyurethane Elastomers with Antistatic Properties and an Influence of Various Additives on Their Physicochemical Properties

**DOI:** 10.3390/molecules26195778

**Published:** 2021-09-24

**Authors:** Szymon Kosiński, Marcin Gonsior, Piotr Krzyżanowski, Iwona Rykowska

**Affiliations:** 1 Department of Analytical Chemistry, Faculty of Chemistry, Adam Mickiewicz University, 61-614 Poznań, Poland; obstiwo@amu.edu.pl; 2 STI Chemsampler, sp. z o. o. sp. k., 61-371 Poznań, Poland; marcin.gonsior@sti-izolacje.pl (M.G.); piotr.krzyzanowski@sti-izolacje.pl (P.K.)

**Keywords:** polyurea, polyurethane, coatings, antistatic additives, ionic liquids, resistance, tensile stress, Fourier transform infrared spectroscopy, scanning electron microscopy

## Abstract

Polyurea is a synthetic high-strength elastomeric material that can be sprayed as a coating over existing structures in order to protect against weathering effects. It is ideal for anti-corrosion protection and is characterized by excellent mechanical properties and adhesion to various surfaces. Further development of this technology may allow obtaining new coatings with improved antistatic properties, which would be an excellent alternative compared to used antistatic epoxy paints. This paper will examine the influence of tetraalkylammonium salt (1), potassium hexafluorophosphate solution (2) and imidazolium-based ionic liquid (3) on the improvement of antistatic properties of the polyurea-polyurethane coatings. In addition, the modified samples were also verified in terms of changes in mechanical properties and the appearance of functional groups other than in the reference sample, as well as surface defects that may arise due to incompatibility of the antistatic additive with the polymer matrix. In order to obtain information about the properties mentioned above, the electrical resistance was determined, the tensile strength and elongation were measured, FT-IR spectra were made, and images were taken with the use of scanning electron microscopy. The conducted research showed that the antistatic properties of the tested hybrid coatings could be improved, but their use may be associated with certain limitations that should be taken into account when designing such materials.

## 1. Introduction

Polyurea is a product of the reaction of two components, isocyanate and a resin mixture, and it exhibits an extremely short “tack-free” time. The isocyanate can exist in two forms: aromatic and aliphatic. In addition, this compound can act as a monomer, a polymer, or a mixture thereof. In almost all commercial formulations, the resin is a homogenous blend of polyetheramine characterized by repeating oxypropylene units in the backbone and chain extender [1,2]. In polyurethanes, hydroxyl-terminated compounds are present instead of amines, while the cross-linking reaction occurs through isocyanate and both amine and hydroxyl-terminated compounds in hybrids [3]. The significant difference between these materials is that there is no need to use a catalyst with pure polyurea. The hybrid reaction, which requires the use of catalysts, becomes uncontrolled when the application is carried out under the ambient condition boundaries relative to the recommended standards. Less frequent crosslinking of bonds may occur at lower temperatures, while it is denser at higher temperatures, affecting the finished product’s mechanical properties [1]. Hybrid materials are, however, less expensive than pure polyurea.

Depending on the need of use, these coatings can be applied manually or with the use of spraying units, which provides a wide range of application possibilities. Polyurea is a material used in many branches of the economy, such as construction, energy, fuels, shipbuilding, waste industry, and ballistic industry. The specific places where it is used are the following: coating layer sealing and securing pipelines; securing coating for steel and reinforced concrete bridges; hydroisolation coating for aquaria, floors, and ceilings for parking spaces; coating layer securing and sealing roof sheathings; securing coating for cargo space for lorries; internal lining for tunnels; coating for containers and pipelines in wastewater treatment plants; elements in nuclear power plants; coatings in entertainment water parks; and waterproof coating for dams. The mentioned applications do not represent all possible uses of the material [4].

The feature that allows such wide use of polyurea is its mechanical resistance, including tensile strength (usually over 20 MPa) and high elongation at break (over 350%) [5,6,7]. The flexibility of the coating is essential due to the possible movement of the protected structure, which may be caused by thermal expansion or vibrations and deformations of its elements. An example of a situation is the thermal interactions in the reservoir in the summer–winter dimension. In some cases, thermal stresses may occur due to short-term effects, such as filling a tank heated by the sun with cold water. Such factors may have a negative impact on the structure to be protected and should be taken into account when selecting the protective coating system so that it has adequate flexibility allowing for crack-bridging [1].

Polyurea is a seamless coating that tightly protects various structures, showing resistance to standing water, and also has the highest class of chemical resistance (class III according to EN 13529), among others, relative to organic acid solutions up to 10%, inorganic acid solutions up to 20%, and alkalis inorganic (except oxidizing) with pH > 8 [7].

In addition to the advantages, polyurea also has disadvantages, one of which is high electrical resistance, which exceeds 10^9^ Ω. The International Electrotechnical Commission in the technical specification IEC/TS 60079-32-1: 2013 [8] defines the criteria for the assessment of floors according to the following requirements:

- Conductive floor with vertical and/or electrical leakage resistance lower than 10^5^ Ω provides full anti-electrostatic protection in all conditions but does not provide protection against electric shock.
- Floor dissipating electrostatic charge with a vertical and/or leakage resistance greater than 10^5^ Ω but less than 10^8^ Ω provides anti-electrostatic protection in all cases, except for processes and technological operations with high dynamics or with materials with high ignition capacity. Such a floor also guarantees adequate protection against electric shock.
- Insulating floor with vertical and/or leakage resistance greater than or equal to 10^8^ Ω does not guarantee anti-electrostatic protection but provides protection against electric shock.

According to these criteria, polyurea should be classified as an insulating material. The document IEC / TS 60079-32-1: 2013 also states that floors in the presence of flammable atmosphere areas should have an electrical leakage resistance of 10^6^ Ω to 10^8^ Ω. However, the subject of static electricity is much more complex, and individual measurement techniques and requirements may differ more or less depending on the case, which may be necessary when designing a system in which the coating is one of the elements.

With its properties but with a sufficiently low electrical resistance value, polyurea can be an excellent alternative to coatings in the systems used [9,10,11,12]. When using the spraying technology, its significant advantage is that it hardens in a few seconds, which allows it to be applied in two immediate passes, providing a 1.5–2 mm layer. Such fast bonding also allows it to be applied on vertical surfaces and even ceilings in a sufficiently thick layer.

Due to the possibility of polyurea modification, its application can be extended by making it a static dissipative or even static conductive material. Such properties are required mostly in electronic and telecommunication industries, computer rooms, hospitals, automotive industries, pharmaceutical factories, aerospace industries [13], refineries, petrochemical industries [14,15,16], solvent storage areas [17], or mining [18]. In the latter case, materials with such properties are particularly needed as wall coatings in road tunnels or as equipment housing materials [19,20]. The static charge that easily builds up on insulating materials may create the conditions for sparking. Usually, it ends up in unpleasant or painful electric shocks, and sometimes it can damage electronic equipment, but it can be a reason for an explosion or fire on a vast scale. Here, the accumulation of electric charge is its separation at the liquid–solid interface resulting from flowing or at the solid–powder-solid interface resulting from friction, collision, and peeling [21]. Appropriate additives for polyurea, which can be ionic liquids (ILs) or inorganic salts, result in new and improved polyurea coatings with static dissipative properties [22]. However, the ability to discharge electric charges in these situations does not mean that the lower the resistance is, the better the material will be for a given purpose. The risk of electrocution from discharge in the electrical network may rise with a too low resistance [23]. The already mentioned ionic liquids are no longer a scientific sensation but are becoming the objects of intensive research even in the industry [24]. They have also been reported to have antistatic properties, rendering them an object of interest in this paper [25,26,27,28,29,30].

## 2. Results and Discussion

### 2.1. Electrical Resistance

The influence of the antistatic additive concentration in the material on the final coating resistance was observed. The results are shown in the figures below. Measurements were not performed for the reference sample at 30% humidity because the obtained results exceeded the measuring range of the device, producing results with errors that are too large. The tendency is the same with each of the additives: the electrical resistance decreases with an increasing antistatic agent, but more or less rapidly; in addition, the effectiveness of lowering the electrical resistance is different for each additive.

**Figure 1:**
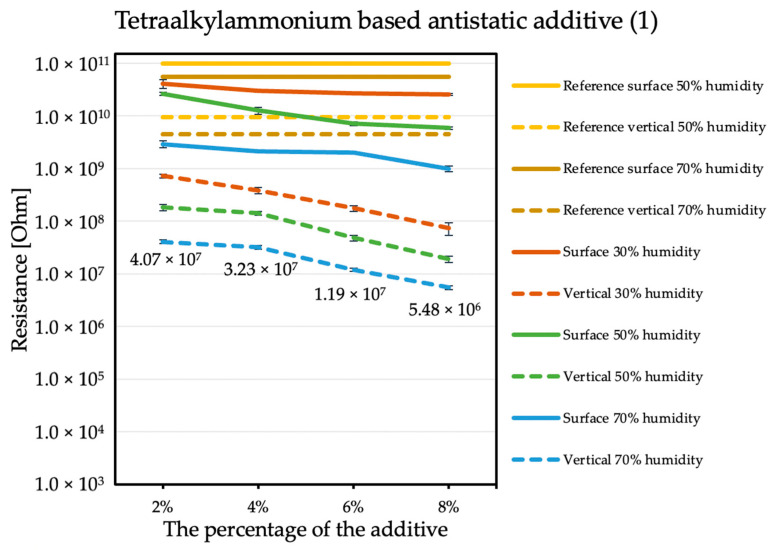
Resistances of coatings with tetraalkylammonium based antistatic additive.

**Figure 2:**
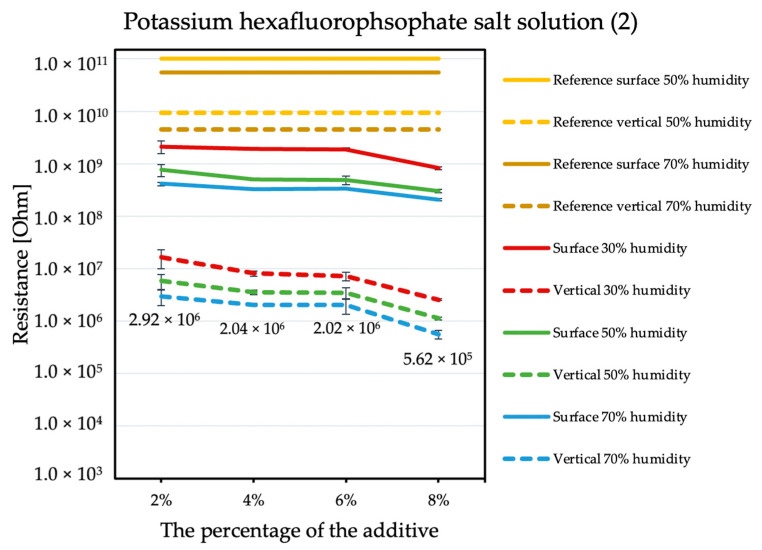
Resistances of coatings with potassium hexafluorophosphate solution additive.

**Figure 3:**
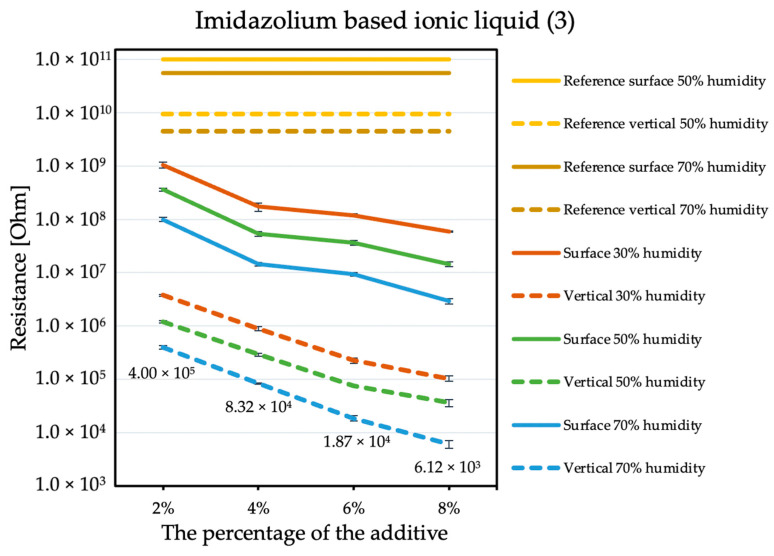
Resistances of coatings with imidazolium based ionic liquid additive.

By comparing the results obtained for the tetraalkylammonium salt additive (Figure 1) with the results obtained for additives number two (Figure 2) and three (Figure 3), the structure of this compound should be explained. In the case of additive no. 1, a long alkyl chain is attached to the tetraalkylammonium cation, and it is known that the increase in alkyl chain affects increasing viscosity. This is the result of an increase in van der Waals forces and a decrease in the mobility of the salt ions, resulting in low ionic conductivity; thus, conductivity decreases with increasing alkyl chain length [31,32]. At this point, it is worth mentioning the viscosity values of the antistatic additives used, which their manufacturers provide in the technical data sheets of their products: viscosity of the first additive is above 3000 mPa·s, while the KPF_6_ solution and the imidazolium-based ionic liquid used in the present study have viscosities of about 15 mPa·s (25 °C). In the case of the second additive, it can be observed that adding it in the amount of 2% causes a decrease in electrical resistance, but increasing its concentration in the first steps does not cause further significant drops. Only when 8% is added, another greater reduction occurs. Nevertheless, even with the addition of 2% of additive no. 2, the electrical resistance results are better than with the addition of 8% of additive no. 1. The no. 3 additive, even at small amounts, significantly improves the antistatic properties of the coating, and each subsequent increase in its concentration in the material increases this ability. Comparing the third additive with the second, it appears that even 2% of additive no. 3 provides an electrical resistance similar or slightly better than that obtained with 8% of additive no. 2. The effectiveness of all the additives used is strongly dependent on the relative air humidity. The highest values of electrical resistance are obtained in a dry environment. However, with increasing humidity, the resistance begins to decrease.

### 2.2. Fourier Transform Infrared Spectroscopy (FT-IR)

**Figure 4:**
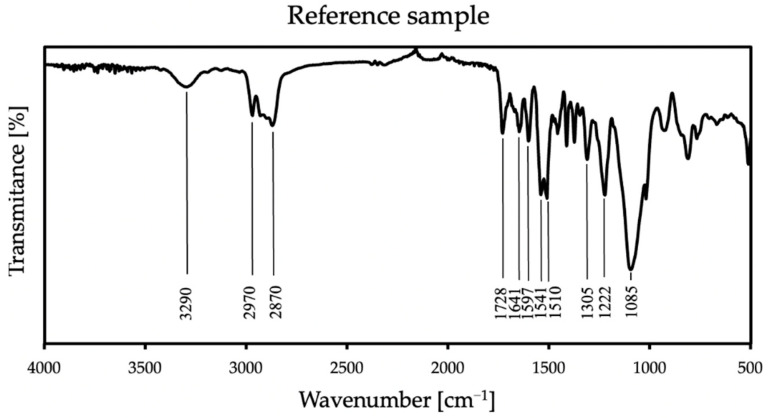
FT-IR spectra of the reference sample.

FT-IR analysis is used to identify organic, inorganic, and polymeric materials. The changes in the pattern of the characteristic absorption bands indicate a change in the composition of the material. FT-IR can be useful for detecting contaminants in a material, finding additives, and identifying degradation and oxidation [33]. In this work, the verification of FT-IR spectra was aimed at checking whether the modification of the material with an antistatic additive would affect the course of the reaction, the formation of new, different bands, or the disappearance of the expected ones. This analysis also helped to exclude product defects that may occur due to improper mixing, which may be caused by air entrainment or differences that are too large in viscosities. The FT-IR results from thirteen samples were obtained and identified based on literature data (Williams and Flemming [34]; Silverstein, Webster, and Kiemle [35]). Figure 4 shows the complete spectral analysis of the reference sample.

**Figure 5:**
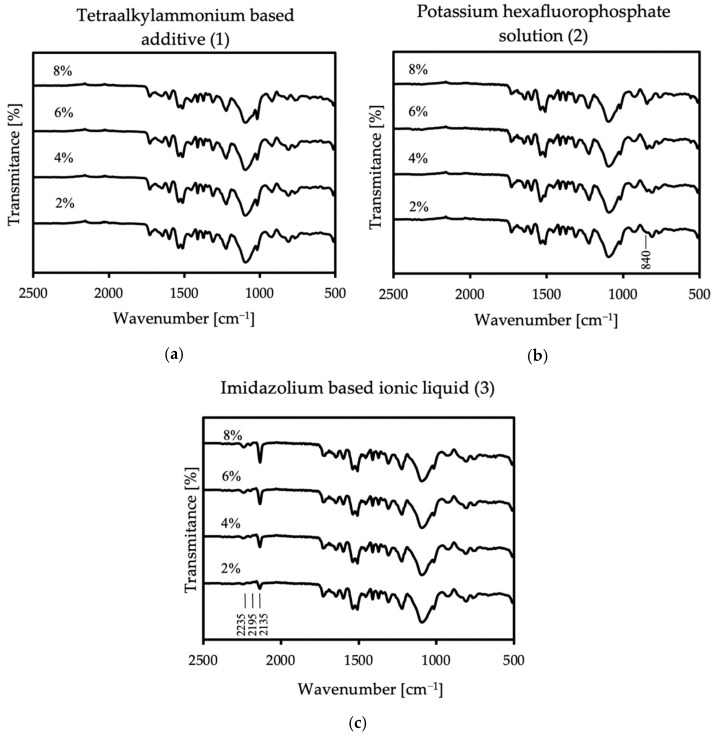
FT-IR spectra of the coatings with all used concentrations of (**a**) no. 1 additive, (**b**) no. 2 additive, and (**c**) no. 3 additive.

In all samples, some characteristic bands appear: at 3290 cm^−1^ is a weak peak of an NH stretch amides, and the FTIR spectrum showed stretching vibrations at 2970 cm^−1^ and 2870 cm^−1^, which stand for CH alkane and aldehyde, respectively [36]. There are also bands at 1728 cm^−1^ from urethane and at 1641 cm^−1^ from urea [37]. The spectra also show NH bend peak at 1597 cm^−1^ and CN stretch alkyl amine peak at 1085 cm^−1^. At 1305 cm^−1^ and 1222 cm^−1^, CO stretch peaks appear—carboxylic and alcohol, respectively. The FT-IR spectrum showed the two asymmetric NO_2_ bands at 1541 cm^−1^ and 1510 cm^−1^. These groups appear because polyurea exposed to UV radiation is susceptible to photodegradation and photo-oxidation on its surface, and it is related to unreacted amino groups. Polyurea subjected to these reactions also slightly changes its color [38]. In the case of all additives, there were no differences in the spectrum compared to the reference sample in the range above 2500 cm^−1^; thus, in Figure 5, only specific wave ranges are presented, showing the spectra of all coatings with each used antistatic additive concentration.

In the case of the first additive, no additional bands appear in the spectrum. However, in the case of additives two and three, additional lines are more intense with increasing antistatic agent. The spectra of the sample containing potassium hexafluorophosphate show a peak at 840 cm^−1^, which stands for PF_6_^−^ anion [39,40]. A very strong peak appears in the sample with imidazolium-based ionic liquid at 2135 cm^−1^, assigned to the asymmetric CN stretch. The next two bands appear at the lengths of 2195 and 2235 cm^−1^, and they are definitely weaker than the previous ones in this area. The first one corresponds to the symmetric CN stretch, while the second one can be assigned to a combination band of the symmetric and asymmetric CN stretching modes. Additive no.3 contains the dicyanamide anion, and the new bands that appeared in the spectrum correspond to its presence [41,42]. The FT-IR analysis shows that antistatic additives may affect the chemical composition of the coating, which may result in a change in some of its properties. The lack of disappearance of the reference spectrum bands in the modified materials proves that the antistatic additives did not adversely affect the course of the reaction itself.

### 2.3. Tensile Tests

An essential factor in the correct determination of the stress-strain properties of the polyurea samples is their proper conditioning. After spraying, polyurea should be aged because this material obtains its exclusive properties over time through its subsequent crosslinking. Each sample consisted of five specimens. The modification of the coatings changed their mechanical properties.

**Figure 6:**
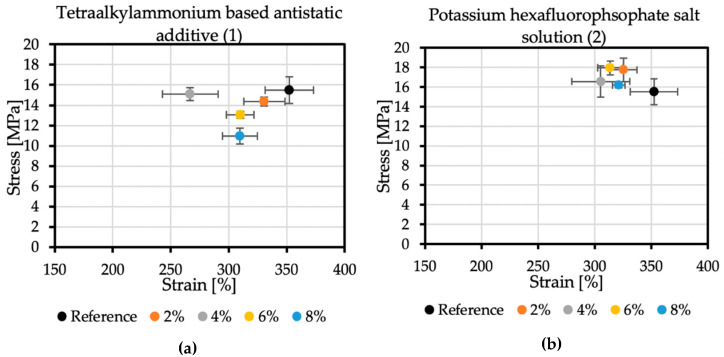

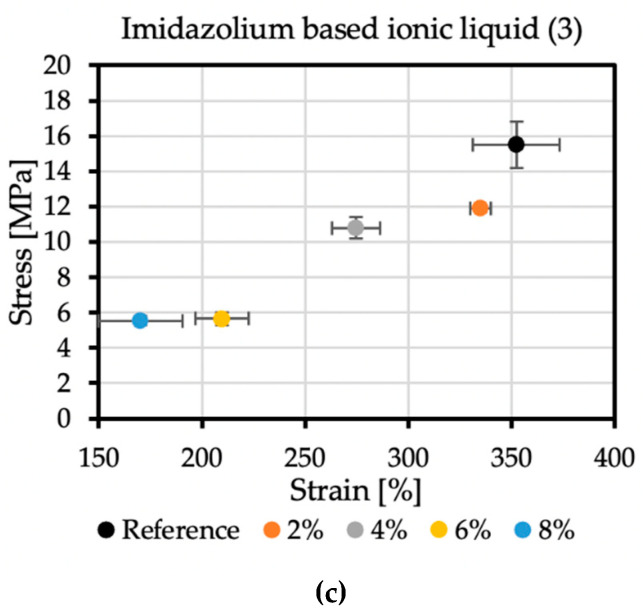
Mechanical properties of samples with (**a**) no. 1 additive, (**b**) no. 2 additive, and (**c**) no. 3 additive.

The reference sample achieved the tensile stress value of 15.5 MPa, while the elongation at break was 352.36%. In the case of additive no. 1 (Figure 6a), increasing its concentration reduces the strength and elongation at break. The result for the 4% concentration, which differs from the trend, may be caused by too early an attempt to remove the sample from the polypropylene plate after spraying. The addition of 8% of antistatic additive resulted in a material that achieved tensile stress of 10.97 MPa and an elongation at break of 309.62%. Additive no. 2 (Figure 6b) is a KPF_6_ solution, and all the obtained results are within a certain range. The strength at break is greater than the reference sample, but the elongation at break is lowered. The addition of 6% of this antistatic additive resulted in a material that achieved tensile stress of 17.95 MPa and an elongation at break of 313.78%. The additive no. 3 (Figure 6c) has a significant impact on the mechanical properties of the sprayed coatings. The addition of 2% of this antistatic additive results in a reduction in tensile stress to 11.9 MPa, resulting in elongation at a break of 335.11%. At the highest concentration of the antistatic additive used, the coating withstands tensile stresses of only 5.54 MPa, while the elongation at break is then 170.38%, which is not even half of the value obtained for the reference sample. The reason for such a significant deterioration of mechanical properties may be a change in the chemical composition of the coating and the incompatibility of the additive with the polymer matrix. The materials made with this antistatic agent showed the weakest tensile tests results, but none of the formulas had been optimized yet.

### 2.4. Scanning Electron Microscopy (SEM)

**Figure 7:**
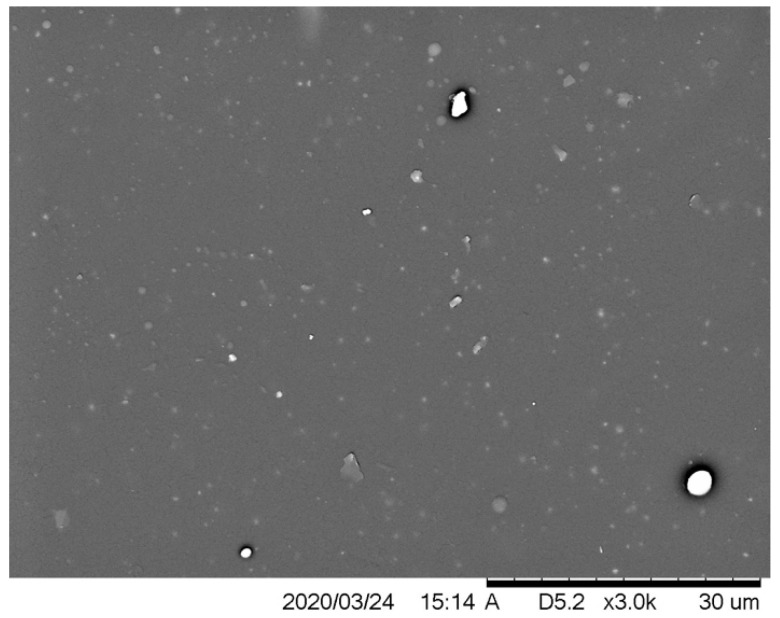
SEM image of the reference sample.

The SEM analysis was performed on the reference sample (Figure 7) and those with modifications with the concentration of 2% and 6% of the antistatic additive. Most samples are structured as expected, relatively smooth and homogenous, and the chosen application technique may cause slight surface irregularities. The images taken excluded the formation of a significant number of microcraters on the surface of the coatings, thanks to which the materials were bonded without breaking their continuity, maintaining the appropriate structure. The antistatic additives could also cause spherical hindrance, which would slow down the curing of the coating, and the isocyanate could start reacting with the moisture in the air, generating an unstable carbamic acid which decomposes into carbon dioxide gas and a primary amine [43]. Carbon dioxide escaping from the as yet not fully cured coating can, thus, damage it by creating holes.

The SEM images were taken in two separate sessions, and the scales placed on them differ slightly. These differences result from the data display settings in the program.

**Figure 8:**
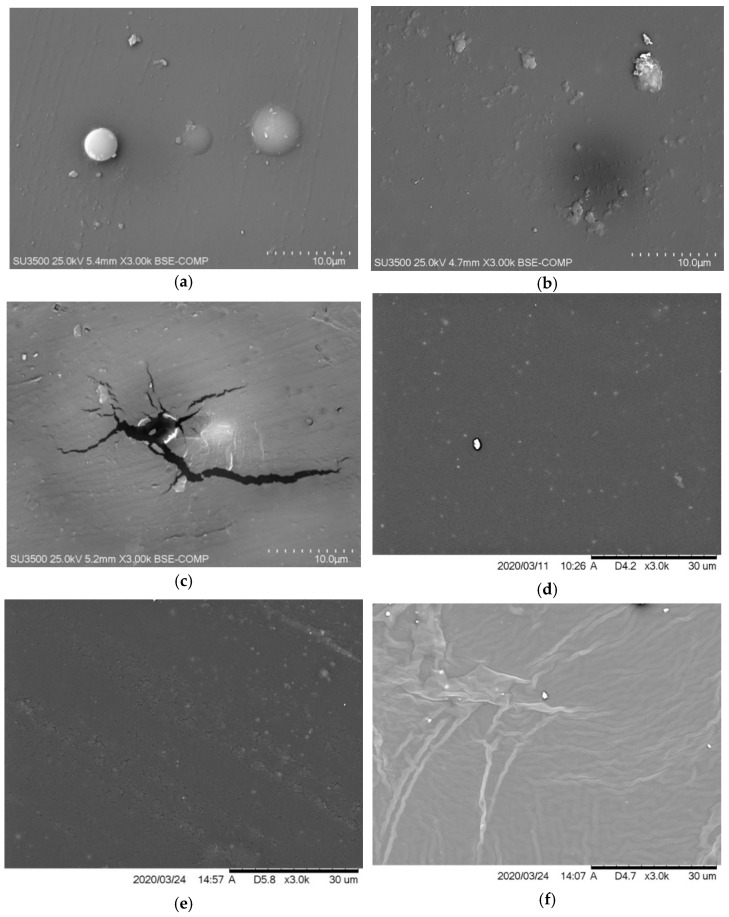
SEM image of the coating containing (**a**) 2% of no. 1 additive, (**b**) 6% of no. 1 additive, (**c**) 2% of no. 2 additive, (**d**) 6% of no. 2 additive, (**e**) 2% of no. 3 additive, and (**f**) 6% of no. 3 additive.

In the case of 2% addition of no. 2 additive (Figure 8c) and 2% addition of no. 3 additive (Figure 8e), a few microcracks can be observed. As found in the literature, the molecules in polyurea after aging could degrade to form micropores, then developing the microcracks [44,45]. It was even observed with the naked eye that only on the samples with additive no. 3, as its concentration increases and under the influence of increased humidity, an oily layer begins to form on the surface of the coatings. Any increase in humidity also causes these coatings to be wetted more. The SEM image of the sample with the content of 6% of additive no. 3 (Figure 8f) also differs from the others. It should also be mentioned here that wiping off this oily layer does not deteriorate the antistatic properties of the coating. Samples with the addition of no. 3 were exposed several times to conditions in which humidity caused the formation of a liquid layer on their surfaces; then the layer was wiped, and the resistance was measured again. Subjecting it to several such cycles leaves the obtained resistance results unchanged. The liquid appearing on the surface may be an ionic liquid. The poor results of the tensile tests could then be explained by the migration of the used additive to the surface and its low compatibility with the polymer matrix. A full explanation of this phenomenon may be the subject of future research.

## 3. Materials and Methods

### 3.1. Samples Preparation

Due to the high reaction rate of pure polyurea, it must be sprayed hydrodynamically. Hybrid coatings were used for the tests because their reaction rates are lower, thanks to which their spraying was possible on a small scale with the use of static mixers. The results obtained in this manner provide an overview of the effectiveness and impact of the antistatic agents used and allow their selection for the design of recipes intended for further tests on industrial spraying units.

All coatings were prepared from commercially available raw materials. The base consisted of the following: part A contains isocyanate—low functional methylene diphenyl diisocyanate (MDI) prepolymer with 15–16% NCO groups from Huntsman (Texas, CA, USA); part B (resin) contains a composition of polyoxyalkylene triol (40–70% parts by weight (pbw)) from PCC Rokita (Brzeg Dolny, Poland), two aromatic diamines (one from Lonza (Basel, Switzerland) (8–14% pbw) and the other from Albemarle (Charlotte, NC, USA) (5–12% pbw)), aliphatic diol (4–8% pbw) from LyondellBasell (Rotterdam, Netherlands), and antistatic agent (except reference sample), catalyst from Vertellus (Indianapolis, IN, USA), defoamer from BYK-Chemie (Wesel, Germany), molecular sieve powder from Grace (Worms, Germany), and pigment paste from Permedia (Lublin, Poland). Antistatic agents were used: tetraalkylammonium salt with ethyl sulphate anion (1), potassium hexafluorophosphate solution (2), and imidazolium-based ionic liquid with dicyanamide anion (3). All antistatic agents were used separately.

**Figure 9:**
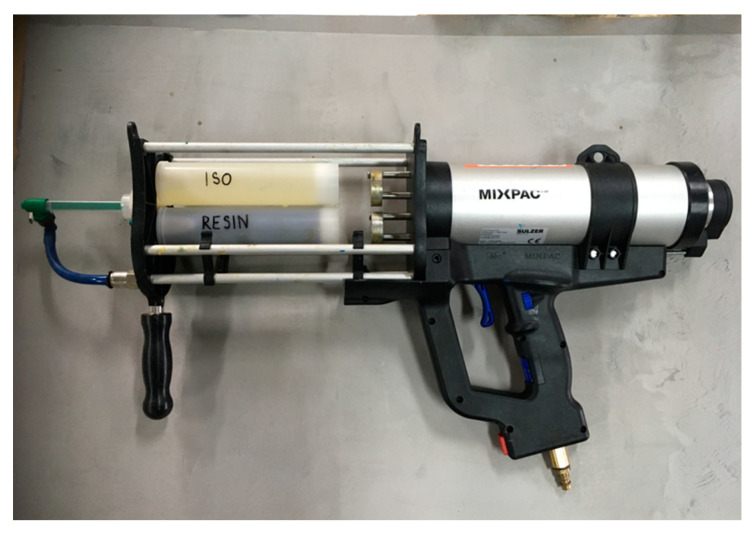
Pneumatic dispenser equipped with a cartridge and static mixer.

The coatings were sprayed with the component volume ratios of 1:1. Adding an antistatic agent requires adjusting the recipe by equalizing the number of functional groups in component B with component A, so that they are the same. Each sample was prepared in a volume of 300 mL by adding all components of Part B to a 500 mL beaker and mixing them with a spiral stirrer for 3 min. Component B, prepared in this manner, and component A were poured into an AF 400-01-10-01 cartridge (200 mL per side) and left for a few minutes for deaeration. The next step of sample preparation was spraying by using a Sulzer mixpac pneumatic dispenser equipped with MFQ 07-18C-06 static mixers (Figure 9). The samples were sprayed onto smooth polypropylene plates, removed after half an hour, left in a room temperature for 12 h, and placed in a climatic chamber at 70 °C for 48 h. As described, the above samples were prepared with concentrations of 2, 4, 6, and 8% for the finished coating for each of the three antistatic additives, and a reference sample. Each sample was approximately 1.5 mm thick and had an area of 0.25 square meters.

### 3.2. Methods

#### 3.2.1. Electrical Resistance

**Figure 10:**
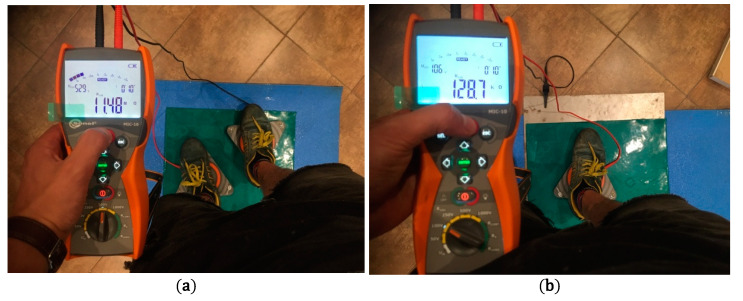
(**a**) Point-to-point and (**b**) vertical resistance measurement.

Measurements were performed with a Sonel MIC-10 device and Sonel PRS-1 probes in accordance with the PN-EN 1081:2001 standard [46]. Before the measurements, the samples were prepared according to the requirements of the standard. Each sample was wiped with a cleaning fluid (ethanol), and then a graphite suspension was applied to the underside of the sample. The materials prepared in this manner were placed in a climatic chamber at a temperature of 40 °C for 96 h. After this step, the conditioning of the samples for the given test started, and it was performed in such a manner that the result reflected the influence of humidity on the electrical resistance values. In order to obtain the result, the sample stayed in the climatic chamber for the next 48 h at a temperature of 23 °C and relative humidity corresponding to the planned test (30%, 50% and 70%). The point-to-point/surface (Figure 10a) and vertical (Figure 10b) resistances were measured. The surface resistance is measured between two tripod electrodes placed at a constant distance of 100 mm on the laid test sample. The vertical resistance is measured between the tripod electrode placed on the surface of the test sample and a metal plate placed under the sample, which works as the base electrode. For resistances lower or equal to 10^6^ Ω, a constant voltage of 100 volts was used, while for resistances greater than 10^6^ Ω, a voltage of 500 volts was used. The resistance was read at 10 s after the voltage was switched on. For areas smaller than 10m^2^, the standard requires at least three measurements. In this work, five measurements of each resistance were made, the obtained results were averaged, and standard deviation was calculated.

#### 3.2.2. Fourier Transform Infrared Spectroscopy (FT-IR)

The Fourier transform infrared spectroscopy measurements were performed by Bruker IFS 113 V spectrophotometer with a diamond cell (the quest single reflection ATR accessory IRAffinity-1) in automatic mode. Scanning conditions were as follows: wavenumber range from 4000 to 500 cm^−1^; resolution 2 cm^−1^. Five scans were performed for each analysis. This test allows verifying the correct mixing of the ingredients and the correct course of the reaction.

#### 3.2.3. Tensile Tests

**Figure 11:**
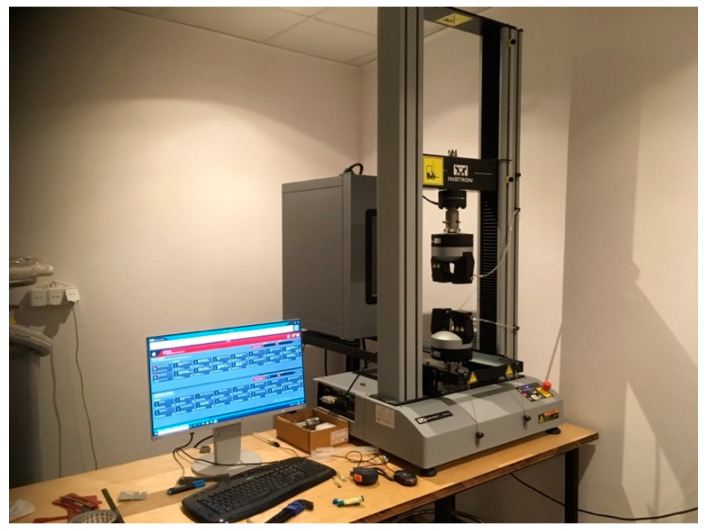
Tensile testing machine.

Tensile tests were performed on a 10 kN Instron 34TM-30 electromechanical tensile testing machine (Figure 11), according to ISO 527-2:2012 standard titled “Plastics—Determination of tensile properties’’ [47]. The tensile test applies a force (pull) to the material, and it measures the sample’s response to stress. This determines how strong the material is and how much it can elongate.

**Figure 12:**
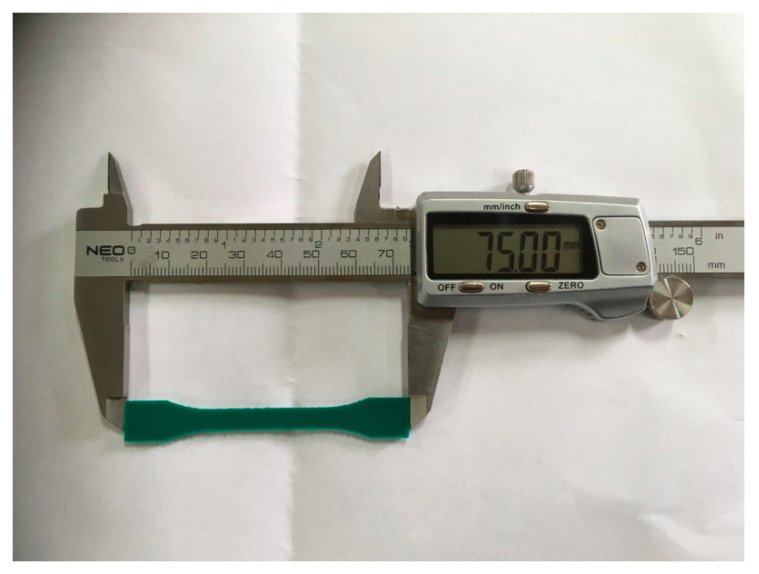
1BA shaped specimen.

Due to the high flexibility of polyurea, 1BA-shaped specimens (Figure 12) were used, which were cut with a milling machine. Each series consisted of five specimens. All tests run up to the failure point with two rates. The first rate was 2 mm/min until the specimen reached 2% elongation. The rate then changed to 100 mm/min. Elongation was measured from the crosshead displacement. Average values and standard deviation were calculated from the obtained tensile strength and elongation at break results.

#### 3.2.4. Scanning Electron Microscopy (SEM)

The surface of the sprayed samples was examined with a scanning electron microscope Hitachi SU3500. The interpretation of the images made it possible to obtain important information on the structure of the sprayed coatings. Microscopic photos made it possible to determine whether there are, for example, material defects such as voids, pores, microcracks, or separation of another phase. This technique was used for investigating the influence of the antistatic additive on the surface appearance. Each sample was photographed and analyzed at the following magnifications: ×100, ×1000, ×3000, ×4000, ×5000. The work presents ×3000 magnification.

## 4. Conclusions

For the purpose of this work, 13 polyurethane-polyurea coatings were made, and the impact of their modifications towards antistatic materials on the physicochemical parameters was verified. The conducted research proved that it is possible to make a hybrid with antistatic and even conductive properties, but it is associated with certain limitations. The effectiveness of the additives used in this work is strongly dependent on the air humidity, which can eliminate them from use in areas where it is very low.

Additive no. 1 is the least effective in reducing electrical resistance. Its significant advantage is the lack of influence on the chemical composition of the material and the lack of surface defects. Increasing its concentration in the coating gradually lowers strength parameters. However, these changes are not very big and are within the limits of expectations.

In the case of additive no. 2, if the coating contains 6% of it, its electrical resistance is slightly lower than that of the coating containing 2%. From an economic point of view, efficiency of the additive is significant. At present, the cost of raw materials needed to produce 1 kg of polyurea is approximately 4.5 €. The antistatic additives used in this work cost 15 €/kg (no. 1), 46 €/kg (no. 2), and 55 €/kg (no. 3). The great advantage of additive no.2 is that it maintains the strength parameters and ensures quite good antistatic efficiency even at just 2% of its addition.

The ionic liquid (no. 3 additive) provides the best antistatic properties, but it is also the most expensive of all the additives used, and each increase in its amount significantly reduces the strength parameters of the tested hybrid coatings. It is also uncertain whether similar phenomena will occur when introducing it to the final recipe on pure polyurea, which will be tested on an industrial spraying unit. Phase separation on the surface of the coating may be particularly undesirable in sterile areas or rooms where people move around due to the risk of slipping. A change in the chemical composition may affect some properties, such as adhesion to various surfaces, resistance to UV radiation, thermal resistance, or chemical resistance. Despite the disadvantages, in the case of a coating containing 2% of additive no. 3, it is possible to obtain outstanding electrical resistance values while maintaining an acceptable reduction in mechanical properties. In this recipe, the phase separation on the surface is negligible, and a large percentage of additive does not exist in the material. The efficiency with little use of it compensates for this loss of mechanical properties.

## Data Availability

The data presented in this study are available on request from the corresponding author. The data are not publicly available due to the fact the patent-application procedure is in progress.

## Sample Availability

Samples of the compounds are not available from the authors.
